# Veterinary neurology residency training in Europe—A survey on preparation and plans

**DOI:** 10.3389/fvets.2024.1487124

**Published:** 2024-10-15

**Authors:** Rita Gonçalves

**Affiliations:** Department of Veterinary Science, Small Animal Teaching Hospital, University of Liverpool, Leahurst, Neston, United Kingdom

**Keywords:** neurology, residency, plans, academia, survey

## Abstract

**Introduction:**

Difficulties in recruitment of veterinary specialists to academia is an ongoing problem for university teaching hospitals. The aim of this study was to determine the proportion of veterinary neurology specialists that plan to work in academia after their residency training and identify the main factors that may influence that decision.

**Methods:**

An electronic survey was distributed to European College of Veterinary Neurology (ECVN) residents in training and those that completed their residencies within the previous 12 months.

**Results:**

Despite similar numbers of residents training in clinical practice and academic institutions, most respondents (79.7%) planned to work in private practice. The most influential factors for deciding future workplace were quality of life, salary, location, and the number of other specialists (with specialists in subjects other than neurology viewed as more important than neurologists) working in the same institution. The most common reasons for not choosing academia were low compensation, excessive bureaucracy, and high administrative workload compared to private clinical practice.

**Discussion:**

Academic institutions need to review support for academic staff and provide stronger mentorship to overcome these problems and ensure provision of high-quality undergraduate teaching in veterinary neurology as well as promoting advancement of the field through basic and applied research.

## Introduction

Neurology is a rapidly evolving field of veterinary medicine. The European College of Veterinary Neurology (ECVN) is a specialty organization that oversees the training and certification process of veterinary neurologists in Europe and as of October 2023 had 252 active diplomates and 108 residents in training. Over the past years there has been a significant growth in the size of this organization, which by comparison, was composed of only 136 diplomates and 45 residents in training in 2013. This expansion, at least partially related to the increased demand for veterinary neurologists and progress in the field, has still not resolved the ongoing shortage of veterinary specialists in academia (1, 2). This is concerning as veterinary neurology specialists in academic institutions are essential for provision of high quality undergraduate teaching in this subject, particularly feared by students which frequently manifest neurophobia (3, 4) and ensure competence in the next generations of veterinarians across the world. These specialists are also crucial for provision of residency training programmes, essential to overcome the shortage of specialists, and for the advancement of the field through basic and applied research.

In human medicine, the American Academy of Neurology conducts triannual surveys of graduating neurology residents to gain insights into training standards and identify opportunities for improvement (5). Similar endeavors to monitor residency programmes in veterinary medicine have not been previously undertaken and there is a need to identify and prioritize areas in need of improvement.

In this study, we conducted an online survey to identify ECVN residents' in training perceptions regarding their level of preparation, future outlook and plans. We aimed to determine how many plan to work in academia and identify the main factors that may influence that decision.

## Materials and methods

A survey designed to assess resident training, outlooks and future plans was developed following review of similar instruments used previously (2, 5, 22). Once the survey was developed, it was reviewed and approved by the executive committee of the ECVN, which consented for contact of the membership. Ethical approval for this study was granted by the Ethics Committee of the University of Liverpool (VREC1325).

In July 2023, the survey was sent via email to 110 ECVN residents in training and also 34 residents that had completed their residencies within the previous 12 months using a commercial online survey software program; survey responses were recorded anonymously via a GDPR-compliant online survey platform, JISC (jisc.ac.uk, Bristol, England). The survey questionnaire comprised of 19 questions collecting demographic data, information regarding the training programme and future career plans (Supplementary material 1). All responses remained anonymous and the software program automatically summarized data and formulated tables and bar graph displays. Free text comments from questions were reviewed for the most common responses.

## Results

### Demographic data

The overall response rate was 41% (59/144). There were similar numbers of female (57.6%) and male (40.7%) respondents and 1.7% identified as other gender. Most respondents were between 30 and 34 years old (52.6%) whilst 27.1% were between 24 and 29 years and 20.3% were 35 years old or more. The vast majority of respondents were of white ethnicity (93.2%) with only 3.4% of mixed or multiple backgrounds and 3.4% Asian. A small number of respondents became a parent during the training period 11.7%, with most acting as the main carer (5/7).

Approximately similar numbers of residents were undertaking or had recently completed the residency training in private practice (52.5%) and in academic institutions (47.5%). The countries where the training programme was based included the United Kingdom (*n* = 25), France (*n* = 9), Italy and Germany (*n* = 7 each), Spain (*n* = 3), and Switzerland (*n* = 2) and one each in the following countries: Canada, Czech Republic, Israel, Netherlands, Slovakia, and Sweden. Training sites varied substantially in the number of residents training at the same time: one resident (23%), two residents (30.5%), three residents (15.3%), and more than three residents (30.5%).

### Experience obtained during training

**Figure 1:**
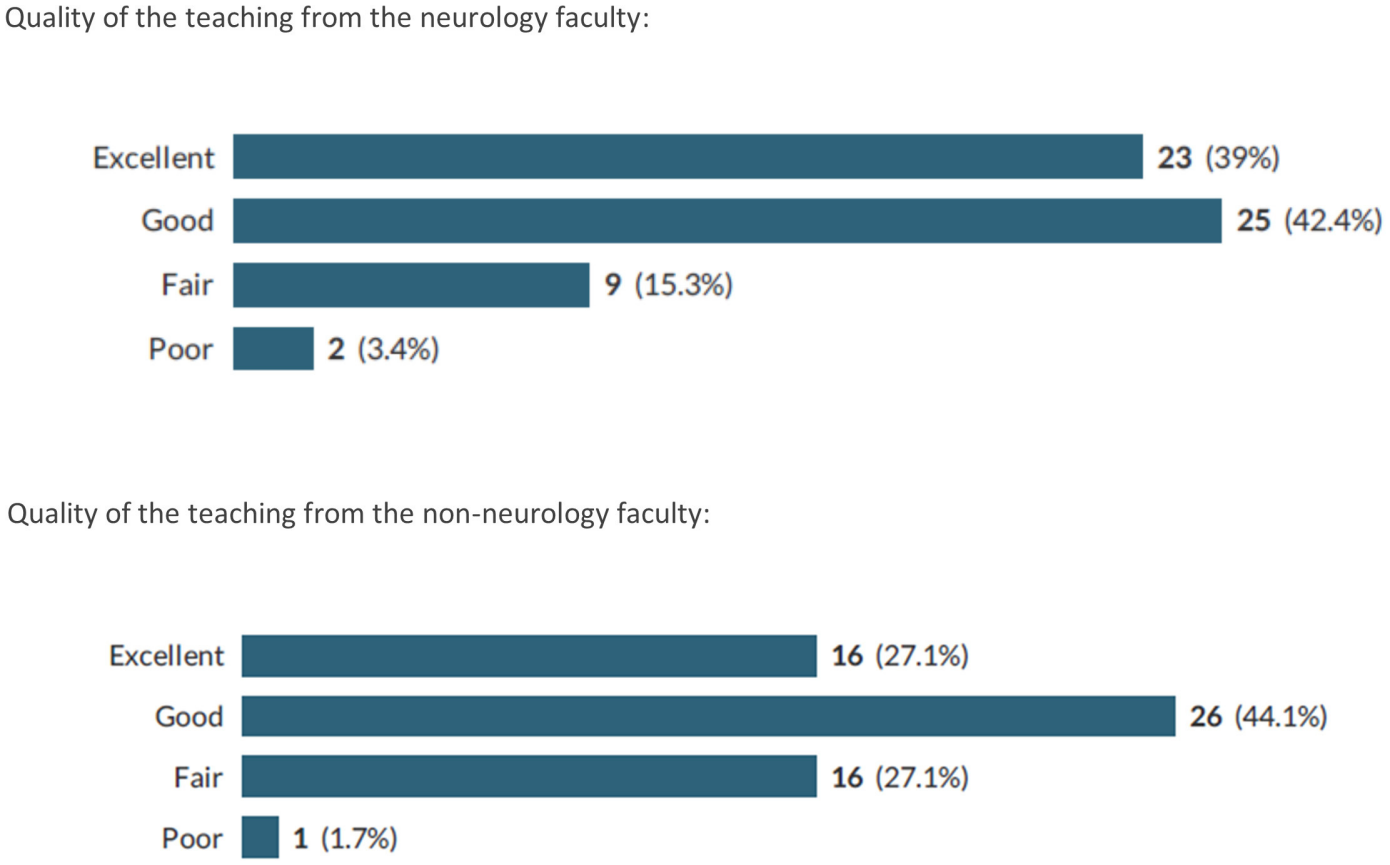
Number (%) of respondents with various perceptions regarding the quality of the teaching received during their residencies.

For most areas of training questioned, respondents felt satisfied with the quality of training received (Table 1). The areas with lowest satisfaction were business skills (with a total of 38.9% dissatisfied), research skills (with a total of 32.2% dissatisfied), basic neurosciences (with a total of 25.4% dissatisfied) and neurosurgery (with a total of 22.1% dissatisfied). The majority of respondents (Figure 1) felt that the quality of teaching offered by their neurology supervisors was good (42.4%) or excellent (39%).

**Table 1:** Summary of responses regarding quality of the training received.

	**Very dissatisfied**		**Somewhat dissatisfied**		**Neutral**		**Somewhat satisfied**		**Very satisfied**	
	**Academia**	**Private practice**	**Academia**	**Private practice**	**Academia**	**Private practice**	**Academia**	**Private practice**	**Academia**	**Private practice**
Patient management	3.4%		0%		5.1%		27.1%		64.4%	
	3.6%	3.2%			7.1%	3.2%	28.6%	25.8%	60.7%	67.7%
Neurosurgery	10.2%		11.9%		5.1%		32.2%		40.7%	
	10.7%	9.7%	7.1%	16.1%	7.1%	3.2%	39.3%	25.8%	35.7%	41.2%
Diagnostic test interpretation	1.7%		1.7%		10.2%		32.2%		54.2%	
	0%	3.2%	3.5%	0%	7.1%	12.9%	32.1%	32.3%	57.1%	51.6%
Research	15.3%		16.9%		18.6%		27.1%		22%	
	7.1%	22.6%	7.1%	25.8%	17.9%	19.4%	39.3%	16.1%	28.6%	16.1%
Basic neurosciences	8.5%		16.9%		16.9%		33.9%		23.7%	
	3.6%	12.9%	17.6%	16.1%	17.9%	16.1%	25%	41.9%	35.7%	12.9%
Undergraduate teaching	10.2%		11.9%		22%		22%		33.9%	
	3.6%	16.1%	7.1%	16.1%	14.3%	29%	21.4%	22.6%	53.6%	16.1%
Postgraduate teaching	8.5%		13.6%		25.4%		28.8%		23.7%	
	3.6%	12.9%	7.1%	19.4%	25%	25.8%	32.1%	25.8%	32.1%	16.1%
Business skills	22%		16.9%		39%		15.3%		6.8%	
	17.9%	25.8%	17.9%	16.1%	42.9%	35.5%	10.7%	19.4%	17.9%	3.2%
Public speaking	13.6%		6.8%		22%		40.7%		16.9%	
	7.1%	19.4%	7.1%	6.5%	32.1%	12.9%	32.1%	48.4%	21.4%	12.9%
Leadership and mentorship skills	16.9%		3.4%		28.8%		32.2%		18.6%	
	10.7%	22.6%	3.6%	3.2%	32.1%	25.8%	42.9%	22.6%	10.7%	25.8%

### Outlook and future plans

**Figure 2:**
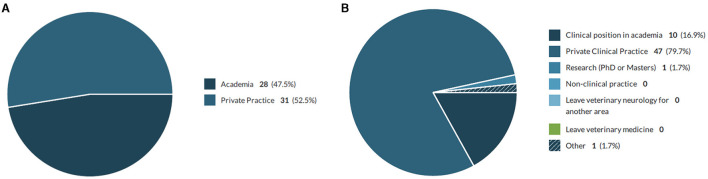
Place of training and plans after residency completion. **(A)** Training institution where respondents are undertaking the residency. **(B)** Plans for work after completing residency training.

The majority of respondents (79.7%) indicated they planned to work in private clinical practice after completion of the residency training (Figure 2); this included 19/28 residents training in academic institutions and 28/31 training in private practice. The most important factors cited as influencing the decision of future work place were quality of life, salary, location, and the number of other specialists (with specialists in subjects other than neurology viewed as more important than neurologists) working in the same institution (Table 2). For the respondents that have chosen not to work in academic institutions, the main reasons justifying this decision were that the salary is too low, there is too much bureaucracy and administrative workload around teaching and research in academia compared to private clinical practice (Table 3).

**Table 2:** Summary of responses regarding plans immediately after the residency training.

	**Not important**		**Somewhat important**		**Very important**	
	**Academia**	**Private practice**	**Academia**	**Private practice**	**Academia**	**Private practice**
Quality of life	0%		5.1%		94.9%	
			3.6%	6.5%	96.4%	93.5%
Clinical caseload	1.7%		61%		37.3%	
	3.6%	0%	60.7%	61.3%	35.7%	38.7%
Academic environment	45.8%		33.9%		20.3%	
	35.7%	54.8%	46.4%	22.6%	17.6%	22.6%
Financial reasons	3.4%		39%		57.6%	
	3.6%	3.2%	35.7%	41.9%	60.7%	54.8%
Location	0%		32.2%		67.8%	
			46.4%	19.4%	53.6%	80.6%
Research opportunities	32.2%		49.2%		18.6%	
	35.7%	29%	46.4%	51.6%	17.6%	19.4%
Opportunity for undergraduate teaching	50.8%		42.4%		6.8%	
	53.5%	48.4%	42.9%	41.9%	3.6%	9.7%
Number of other neurologists working on site	10.2%		54.2%		35.6%	
	14.3%	6.5%	42.9%	64.5%	42.9%	29%
Other services available on site	0%		25.4%		74.6%	
			28.6%	22.6%	71.4%	77.4%
Parental leave	45.8%		33.9%		20.3%	
	46.4%	45.1%	32.1%	35.4%	21.4%	19.5%

**Table 3:** Summary of responses regarding reasons for not wanting to work in academia.

	**Strongly disagree**		**Somewhat disagree**		**Somewhat agree**		**Strongly agree**	
	**Academia**	**Private practice**	**Academia**	**Private practice**	**Academia**	**Private practice**	**Academia**	**Private practice**
The salary is too low	0%		16.7%		37.5%		45.8%	
			5.6%	23.3%	22.2%	46.7%	72.2%	30%
Academic environments are too bureaucratic	2.1%		16.7%		45.8%		35.4%	
	0%	3.3%	22.2%	13.3%	38.9%	50%	38.9%	33.4%
I do not want to teach undergraduate students	52.1%		27.1%		14.6%		6.3%	
	50%	53.3%	33.3%	23.3%	11.1%	16.7%	5.6%	6.7%
I do not want to do research	35.4%		43.8%		16.7%		4.2%	
	38.9%	33.3%	38.9%	46.7%	16.7%	16.7%	5.5%	3.3%
Too much administrative work around teaching and research	4.2%		31.3%		41.7%		22.9%	
	5.6%	3.3%	27.7%	33.3%	38.9%	4.3%	27.7%	20%
No available positions where I wanted to work	27.1%		37.5%		22.9%		12.5%	
	27.7%	26.7%	38.9%	36.7%	16.7%	26.7%	16.7%	10%
There is lack of good mentoring and professional guidance for support on this path	22.9%		33.3%		33.3%		10.4%	
	33.3%	16.7%	27.7%	36.7%	33.3%	33.3%	5.6%	13.3%

**Figure 3:**
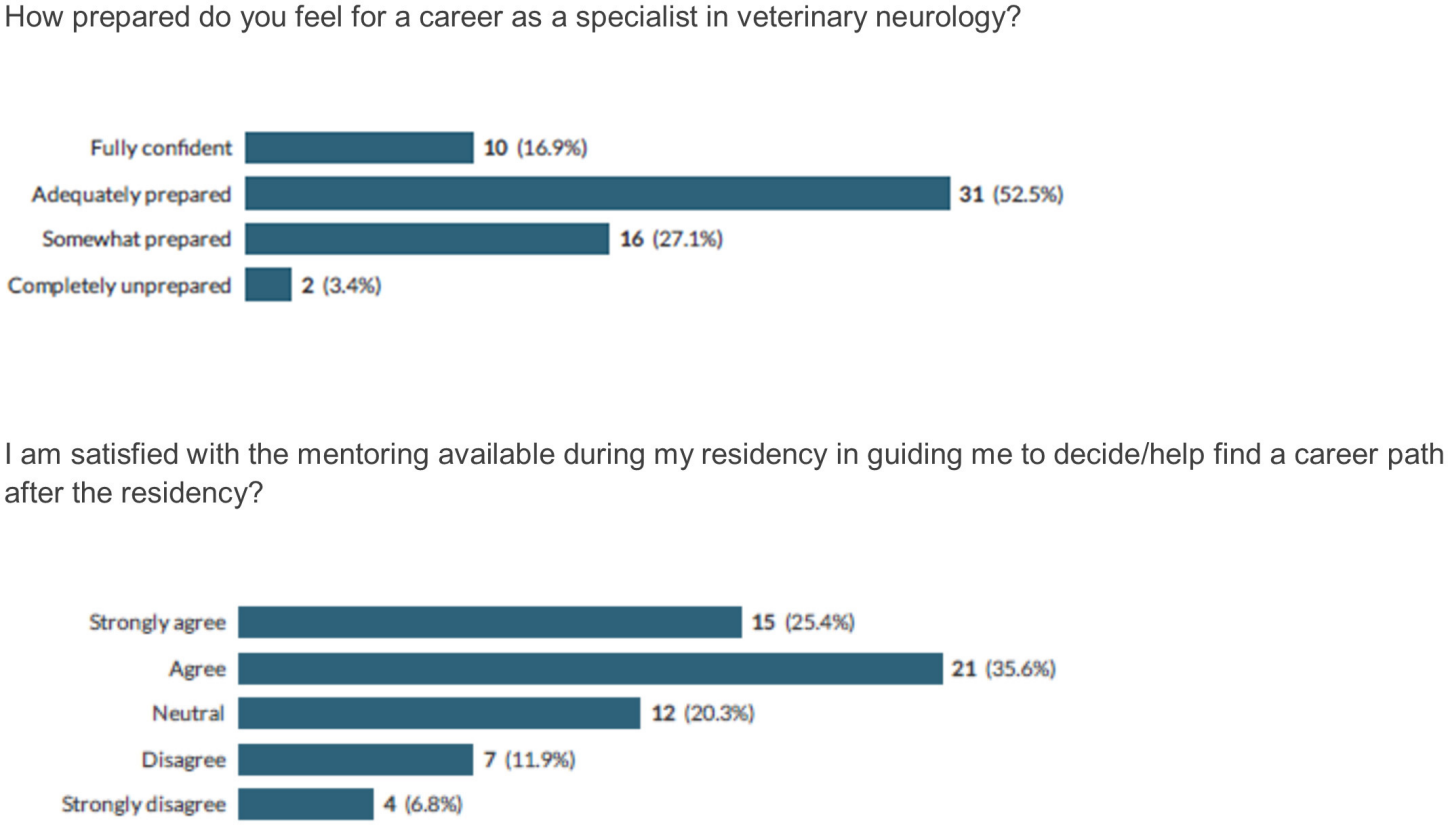
Respondents' perceptions regarding their preparedness for a career as a veterinary neurology specialist and satisfaction with their mentoring in choosing a career path.

Most respondents (69.4%) felt fully or adequately prepared for a career as a specialist in veterinary neurology and most (61%) were satisfied with the mentoring available during the residency in guiding their future career path (Figure 3).

## Discussion

The present study provides a snapshot of the current veterinary neurology specialist training in Europe. Our results show that despite the training centers being similarly divided between private clinical practice and academic institutions, most respondents planned to work in private practice after completion of the training. The most common reasons for not choosing academia was the belief that compensation is too low, that the work would involve too much bureaucracy and that the administrative workload is too high compared to private clinical practice.

Our demographic data suggest that the majority of respondents were of white ethnicity, highlighting the limited diversity commonly reported within the veterinary profession (6–8). Strategies to prioritize diversity as a core value for recruitment in veterinary medicine have been in place in the United States for several years (9, 10) but similar policies are less frequent in Europe. It also highlighted that only a small percentage of responders became a parent during the training period despite most (72.9%) being 30 years or older. Unfortunately, due to the confidential collective analysis of the responses, it is not possible to identify the gender of those that became parents. Previous studies have identified that surgical and neurology trainees often leave residencies when considering becoming a parent because of the stigma associated with pregnancy during training and dissatisfaction with parental leave options (11–13), with parenthood being one of the factors contributing to attrition in surgical specialties. It would be useful to start a discussion regarding the support for parental leave during specialty training programmes in veterinary medicine in order to promote retention of colleagues but also to increase productivity and job satisfaction. Despite the predominance of female residents (58%), this is likely lower than the proportion of female graduates which has been reported as 79% in the US (14) and 76% in the UK (15), but the number of female veterinary graduates across Europe is unknown. The proportion of women applying for veterinary specialty residencies was lower than the overall proportion of female graduates in the US but when applying, women were not less likely to have successful applications than men (14).

Most respondents were satisfied by the quality of teaching offered by their neurology supervisors but almost 40% were not fully satisfied with the mentoring in terms of guidance when deciding a future career path. Mentorship is usually described as the process through which an experienced person guides another in developing skills and knowledge for their professional development (16). This may be considered particularly important in the medical and veterinary professions, where varied career paths are possible including varied combinations of professional roles such as clinical practice, teaching, supervision, administrative and research roles. Previous research has shown that mentors can significantly contribute to the development of mentees' teaching and clinical skills, research outputs as well as their networking abilities and career management (17). In view of our findings, the development of mentoring programmes, both by the training institutions and the ECVN, should be considered so it could enhance the residents capability of achieving their desired career outcomes. Almost a third of respondents reported not feeling adequately prepared for a career as a specialist in veterinary neurology. Unfortunately, our data does not allow determining the year of training each responder was in but it is likely that those not feeling prepared were in the earlier stages of their training.

Certain topics within veterinary neurology were highlighted as areas where teaching should be strengthened, including research and business skills (approximately one third of respondents showed dissatisfaction with the training in these areas). Similar concerns have also been highlighted in different residency training programmes in human medicine and attempts to address these by strengthening the curricula have been undertaken over the years (5, 18–20). Parallel targeted education efforts during the veterinary neurology residency to mitigate these gaps in the training would be beneficial and should be promoted by both the ECVN and training institutions. Basic neuroscience and neurosurgical training were also topics where the teaching provided was associated with dissatisfaction in approximately one quarter of respondents. Both topics are of crucial importance in veterinary neurology and it is essential that steps are taken to strengthen the training provided in these areas.

After completing a specialty training, veterinarians have a number of career paths to pursue but only one respondent reported planning to undertake a research role after the residency and none was interested in industry. It was not surprising that the majority of respondents plans to work in private clinical practice after completion of the training programme, as recruitment and retention of clinical veterinary specialists in academia has previously been shown to be a problem (1, 2, 21–23). Most people that enter specialty training likely do this because they want to be clinicians and practice their specialty so this could be one of the reasons why the majority chose a path in specialty practice. The most important factor for choosing a future in private clinical practice was the difference in salary compared to academia. This concern had been previously reported in other veterinary specialties (2, 21, 22), where in some instances salaries were reported to double when moving from academia to private practice (22). The impact of the job market and number of employment opportunities available after training likely contribute to determination of salary rates and currently, there is still a significant demand for veterinary neurology specialists which will likely be driving the disparity in salary rates. Bureaucracy and high administrative workload around teaching and research were also highlighted as significant considerations associated with academia and this had been highlighted in previous studies (21, 24). Whilst addressing the difference in compensation would likely prove challenging for academic institutions, reducing bureaucracy and setting realistic targets for teaching and research outputs of academic staff should be achievable. Such changes require support from academic management and the provision of strong mentorship to provide information about academic career paths and demonstrate the benefits and opportunities that exist in academia. Having had a positive residency training experience has previously been shown to have a positive effect on desiring a career in academia (25) and effective mentoring has been identified as one of the most influential reasons to pursue an academic career (24, 26) so academic institutions should prioritize the quality of their programmes and develop mentorship training to ensure staff retention. Interestingly, most respondents were not reluctant to undertake undergraduate teaching and rated the opportunity to undertake research as important, suggesting that, with some changes to professional rewards and workplace culture, academic attrition rates could decrease. The creation of post-residency clinical research training fellowships (often 3-year posts), similar to what is widely available in human neurology and neurosurgery specialties, should be considered by academic training institutions as this would likely attract specialists with an interest in research. In order to better understand possible shifts in the decision making process of future career paths during the residency training itself, future longitudinal studies should be considered to establish if this changes with experience and duration of the training already undertaken. This would also allow investigating if certain interventions or training opportunities should be made available to help this process. There are several challenges associated with the transition from clinical practice to academia, often related to a lack of structure and processes to support this transition period. In other healthcare professions, this transition has been reported to cause anxiety and fear of being considered an inadequate teacher and of losing their clinical skills (27, 28). To mitigate these concerns, appropriate training and structure induction programmes are required.

There are different benefits to working in academia and private practice. In academia, there are more opportunities to undertake original research, often grant-funded. Academics participate in education and training of undergraduate and postgraduate (interns and residents) students and often receive mentorship from more senior colleagues in order to further their research interests and career goals. There are often several non-salary benefits such as scheduled academic time and the opportunity to participate in administration and policy. In private practice, the opportunity to undertake 100% clinical practice with no expectations to produce research is highly valued by many. There is greater salary compensation and often more flexibility in the work pattern. There are also opportunities to explore economic and business aspects in terms of management of a growing private practice. This survey focused on identifying the main reasons for academic attrition so the questions were biased toward understanding the reasons why respondents do not intend to pursue careers in this area. This poses a limitation into understanding whether respondents also identified similar disadvantages to working in private practice, or different ones that were not asked, such as pressure to generate income or lack of autonomy.

## Conclusion

In conclusion, despite similar numbers of residents training in clinical practice and academic institutions, most respondents planned to work in private practice. The most common reasons for veterinary neurology residents not choosing academia were low compensation, excessive bureaucracy and high administrative workload compared to private clinical practice. Academic institutions need to review support for academic staff and provide stronger mentorship to overcome these problems and ensure provision of high quality undergraduate teaching in veterinary neurology as well as promoting advancement of the field through basic and applied research.

## Data Availability

The original contributions presented in the study are included in the article/Supplementary material, further inquiries can be directed to the corresponding author.
