# Supplementary material for: Veterinary neurology residency training in Europe—A survey on preparation and plans
Source: Front Vet Sci. 2024 Oct 15;11:1487124. doi: 10.3389/fvets.2024.1487124 (PMC11518817; doi:10.3389/fvets.2024.1487124)
Supplement: Supplementary file 1 [file Table_1.docx]

Supplementary Material

**2023 ECVN Resident survey frequencies**

1. How satisfied are you with the training received during your residency on the following:
   1. Preparing you adequately for patient management

64.4% very satisfied

27.1% somewhat satisfied

5.1% neutral

0% somewhat dissatisfied

3.4% very dissatisfied

- 1. Preparing you adequately for neurosurgery

40.7% very satisfied

32.2% somewhat satisfied

5.1% neutral

11.9% somewhat dissatisfied

10.2% very dissatisfied

- 1. Preparing you adequately for diagnostic test interpretation

54.2% very satisfied

32.2% somewhat satisfied

10.2% neutral

1.7% somewhat dissatisfied

1.7% very dissatisfied

- 1. Preparing you adequately for research

22% very satisfied

27.1% somewhat satisfied

18.6% neutral

16.9% somewhat dissatisfied

15.3% very dissatisfied

- 1. Providing teaching on basic neurosciences

23.7% very satisfied

33.9% somewhat satisfied

16.9% neutral

16.9% somewhat dissatisfied

8.5% very dissatisfied

- 1. Preparing you for undergraduate (vet students) teaching

33.9% very satisfied

22% somewhat satisfied

22% neutral

11.9% somewhat dissatisfied

10.2% very dissatisfied

- 1. Preparing you for postgraduate (vets) teaching

23.7% very satisfied

28.8% somewhat satisfied

25.4% neutral

13.6% somewhat dissatisfied

8.5% very dissatisfied

- 1. Providing you with business skills

6.8% very satisfied

15.3% somewhat satisfied

39% neutral

16.9% somewhat dissatisfied

22% very dissatisfied

- 1. Preparing you for public speaking

16.9% very satisfied

40.7% somewhat satisfied

22% neutral

6.8% somewhat dissatisfied

13.6% very dissatisfied

- 1. Providing you with leadership and mentorship skills

18.6% very satisfied

32.2% somewhat satisfied

28.8% neutral

3.4% somewhat dissatisfied

16.9% very dissatisfied

1. Overall, what was the quality of teaching from the neurology faculty in the institution of your residency?

39% Excellent

42.4% Good

15.3% Fair

3.4% Poor

1. Overall, what was the quality of teaching from the non-neurology faculty in the institution of your residency?

27.1% Excellent

44.1% Good

27.1% Fair

1.7% Poor

1. How satisfied are you with the research opportunities and support available during the residency?

20.3% very satisfied

37.3% somewhat satisfied

18.6% neutral

15.3% somewhat dissatisfied

8.5% very dissatisfied

1. How satisfied are you with the teaching provided during clinical rotations required during your residency training?
   1. Internal Medicine

31% very satisfied

36.2% somewhat satisfied

5.2% neutral

3.4% somewhat dissatisfied

0% very dissatisfied

24.1% has not undertaken this rotation yet

- 1. Diagnostic Imaging

41.4% very satisfied

17.2% somewhat satisfied

6.9% neutral

3.4% somewhat dissatisfied

0% very dissatisfied

31% has not undertaken this rotation yet

- 1. Anaesthesia and/or Emergency and Critical Care

31.6% very satisfied

26.3% somewhat satisfied

8.8% neutral

1.8% somewhat dissatisfied

0% very dissatisfied

31.6% has not undertaken this rotation yet

- 1. General pathology

25.9% very satisfied

17.2% somewhat satisfied

5.2% neutral

12.1% somewhat dissatisfied

1.7% very dissatisfied

37.9% has not undertaken this rotation yet

- 1. Ophthalmology

37.9% very satisfied

19% somewhat satisfied

10.3% neutral

3.4% somewhat dissatisfied

1.7% very dissatisfied

27.6% has not undertaken this rotation yet

1. What are your plans immediately after your residency?

16.9% Clinical position in academia

79.7% Private clinical practice

1.7% Research (PhD or Masters)

1.7% Unsure

1. How important are the following factors in deciding your plans for immediately after your residency
   1. Quality of life

94.9% very important

5.1% somewhat important

0% not important

- 1. Clinical caseload

37.3% very important

61% somewhat important

1.7% not important

- 1. Academic environment

20.3% very important

33.9% somewhat important

45.8% not important

- 1. Financial reasons

57.6% very important

39% somewhat important

3.4% not important

- 1. Location

67.8% very important

32.2% somewhat important

0% not important

- 1. Research opportunities

18.6% very important

49.2% somewhat important

32.2% not important

- 1. Opportunity for undergraduate teaching

6.8% very important

42.4% somewhat important

50.8% not important

- 1. Number of other neurologists working on site

35.6% very important

54.2% somewhat important

10.2% not important

- 1. Other services available on site (e.g. internal medicine, ECC, ophthalmology)

74.6% very important

25.4% somewhat important

0% not important

- 1. Parental leave

20.3% very important

33.9% somewhat important

45.8% not important

1. If you have not chosen a path in an academic institution, what reasons mostly contributed to this?
   1. The salary is too low

45.8% strongly agree

37.5% somewhat agree

16.7% somewhat disagree

0% strongly disagree

- 1. Academic environments are too bureaucratic

35.4% strongly agree

45.8% somewhat agree

16.7% somewhat disagree

2.1% strongly disagree

- 1. I do not want to teach undergraduate students

6.3% strongly agree

14.6% somewhat agree

27.1% somewhat disagree

52.1% strongly disagree

- 1. I do not want to do research

4.2% strongly agree

16.7% somewhat agree

43.8% somewhat disagree

35.4% strongly disagree

- 1. There is too much administrative work around teaching and research

22.9% strongly agree

41.7% somewhat agree

31.3% somewhat disagree

4.2% strongly disagree

- 1. There were no available positions where I wanted to work

12.5% strongly agree

22.9% somewhat agree

37.5% somewhat disagree

27.1% strongly disagree

- 1. There is lack of good mentoring and professional guidance for support on this path

10.4% strongly agree

33.3% somewhat agree

33.3% somewhat disagree

22.9% strongly disagree

1. How prepared do you feel for a career as a specialist in veterinary neurology (taking into account how much of your residency you have completed so far)?

16.9% Fully confident

52.5% Adequately prepared

27.1% Somewhat prepared

3..4% Completely unprepared

1. I am satisfied with the mentoring available during my residency in guiding me to decide/help find a career path after the residency?

25.4% strongly agree

35.6% agree

20.3% neutral

11.9% disagree

6.8% strongly disagree
